# Supplementary material for: Pegivirus Detection in Cerebrospinal Fluid from Patients with Central Nervous System Infections of Unknown Etiology in Brazil by Viral Metagenomics
Source: Microorganisms. 2023 Dec 22;12(1):19. doi: 10.3390/microorganisms12010019 (PMC10818654; doi:10.3390/microorganisms12010019)
Supplement: Supplementary file 1 [file microorganisms-12-00019-s001.zip › Supplementary Material and Methods S1.R1.pdf]

Supplementary Material and Methods S1

Screening assay for Group 1 and Group 2 Pegiviruses by RT-PCR

Briefly, Pegiviruses involved RNA extracted from 300uL individual CSF using the KingFisher™ Flex System (Thermo Scientific™), followed by first-strand cDNA synthesis using SuperScript™ III Reverse Transcriptase with random primers (Invitrogen™). Nested PCRs were conducted with Platinum Platinum™ Taq DNA Polymerase and specific primers for HHpgV-1/group 2 primers and HPgV/group 1 primers. The PCR conditions included 40 cycles of 18 seconds at 94°C, 21 seconds at 50°C, and 60 seconds at 72°C, followed by a final extension of 5 minutes at 72°C. The second round of PCR used 2uL the first-round template under identical thermal conditions [1].

Real-Time PCR for HPgV Detection

For the detection of HPgV, 5 µL of viral RNA extracted from CSF were employed using the TaqMan™ Fast Virus One-Step Master Mix (Applied Biosystems, Foster City, CA, USA) following the manufacturer's guidelines for fast RT-PCR systems, with a final volume of 20 µL. The primers and hydrolytic probe used (IDT™) target a 176 bp fragment within the NS3 protein domain of the virus (Table 1) and were designed to specifically anneal to genotypes 1, 2, 3, 4, and 5 of HPgV. The qRT-PCR reaction was conducted on the StepOnePlus instrument (Applied Biosystems), and the run conditions are depicted in Table 2. Data from each run were analyzed using the StepOne v. 2.3 software (Applied Biosystems) [2].

Table 1 Primers and hydrolytic probe used for amplification of HPgV RNA.

Name	Primer Sequence (5′-3′)	Fragment (bp)
HPgVF	TTACGACGACTGCCCTTACA	176
HPgVR	ACAGTGTTTCCCGGCACAT	
Probe TaqMan®	FAM/AAAAGT(I)CGCGGCGTCAA/BHQ(1)	108-68

Table 2 Thermocycling profile

**Step**	**Cycle**	**Temperature**	**Time**
Reverse Transcription	1	50 ºC	5 minutes
Inactivation and Polymerase Activation	1	95 ºC	20 seconds
Polimerase Chain reaction	40	95 ºC	3 seconds
		60 ºC(1)	30 econds

1. Bonsall, D.; Gregory, W.F.; Ip, C.L.; Donfield, S.; Iles, J.; Ansari, M.A.; Piazza, P.; Trebes, A.; Brown, A.; Frater, J.; Pybus, O.G.; Goulder, P.; Klenerman, P.; Bowden, R.; Gomperts, E.D.; Barnes, E.; Kapoor, A.; Sharp, C.P.; Simmonds, P. Evaluation of Viremia Frequencies of a Novel Human Pegivirus by Using Bioinformatic Screening and PCR. *Emerg Infect Dis* **2016**, *22(4*), 671-8. doi: 10.3201/eid2204.151812.

2. Dias J.Z.C. Influência do Pegivirus humano (HPgV) na medula óssea: impacto clínico e tropismo viral. Tese de Doutorado, Faculdade de Medicina, Universidade de São Paulo, São Paulo, **2018.**
