# Supplementary material for: Pegivirus Detection in Cerebrospinal Fluid from Patients with Central Nervous System Infections of Unknown Etiology in Brazil by Viral Metagenomics
Source: Microorganisms. 2023 Dec 22;12(1):19. doi: 10.3390/microorganisms12010019 (PMC10818654; doi:10.3390/microorganisms12010019)
Supplement: Supplementary file 1 [file microorganisms-12-00019-s001.zip › Supplementary Table S1 Pegivirus PoliprotR1.pdf]

Supplementary Table S1. Data on HPgV reference strains obtained from GenBank for use in phylogenetic analysis.

Weblink	NCBI accession number	ID-sequence	Country	Year	Sample	Reference
-	OR639931	IAL-425/BRA/SP/2019	Brazil	2019	Cerebrospinal Fluid	This study
https://www.ncbi.nlm.nih.gov/nucleotide/D87255	D87255	HGV-Iw	Japan	1996	N.A.	Shao et al. 1996[1]
https://www.ncbi.nlm.nih.gov/nucleotide/AF081782	AF081782	pHGVqz	China	1999	N.A	Xiang et al. 2000[2]
https://www.ncbi.nlm.nih.gov/nucleotide/MK291245	MK291245	H.sapiens-wt/USA/GBVC-SM2	USA	2019	Blood/Plasma	Unpublished
https://www.ncbi.nlm.nih.gov/nucleotide/LT009481	LT009481	56330227	South Africa	2016	N.A	Bonsall et al. 2016[3]
https://www.ncbi.nlm.nih.gov/nucleotide/AY196904	AY196904	765	USA	2003	Human Plasma	George et al. 2003[4]
https://www.ncbi.nlm.nih.gov/nucleotide/MZ420568	MZ420568	HPgV_SP11	Espana	2022	Blood	Cebriá-Mendoza et al. 2021[5]
https://www.ncbi.nlm.nih.gov/nucleotide/MZ420569	MZ420569	HPgV_SP13	Espana	2022	Blood	Cebriá-Mendoza et al. 2021[5]
https://www.ncbi.nlm.nih.gov/nucleotide/LT009487	LT009487	89860237	United kingdom	2016	N.A.	Bonsall et al. 2016[3]
https://www.ncbi.nlm.nih.gov/nucleotide/MZ420575	MZ420575	HPgV_SP38	Espana	2022	Blood	Cebriá-Mendoza et al. 2021[5]
https://www.ncbi.nlm.nih.gov/nucleotide/NC_001710	NC_001710	2700757	USA	1996	Plasma	Linnen et al. 1996[6]
https://www.ncbi.nlm.nih.gov/nucleotide/LT009485	LT009485	56330265	United Kingdom	2016	N.A.	Bonsall et al. 20163
https://www.ncbi.nlm.nih.gov/nucleotide/MZ420570	MZ42057	HPgV_SP19	Espana	2022	Blood	Cebriá-Mendoza et al. 2021[5]
https://www.ncbi.nlm.nih.gov/nucleotide/MZ420576	MZ420576	HPgV_SP44	Espana	2022	Blood	Cebriá-Mendoza et al. 2021[5]
https://www.ncbi.nlm.nih.gov/nucleotide/MH053118	MH053118	JD2B15C	France	2018	Blood Donor	Jordier et al. 2019[7]
https://www.ncbi.nlm.nih.gov/nucleotide/MK291244	MK291244	H.sapiens-wt/USA/GBVC-SM3	USA	2019	Blood/Plasma	Unpublished
https://www.ncbi.nlm.nih.gov/nucleotide/LT009479	LT009479	89859262	United Kingdom	2016	N.A.	Bonsall et al. 2016[3]
https://www.ncbi.nlm.nih.gov/nucleotide/LT009478	LT009478	89859249	United Kingdom	2016	N.A.	Bonsall et al. 2016[3]
https://www.ncbi.nlm.nih.gov/nucleotide/AB003289	AB003289	CG01BD	Japan	2007	Human Serum	Takahashi et al. 1997[8]
https://www.ncbi.nlm.nih.gov/nucleotide/AF031827	AF031827	HGV-T55875	USA	2002	Blood Donor	Bukh et al. 1998[9]
https://www.ncbi.nlm.nih.gov/nucleotide/MZ420574	MZ420574	HPgV_SP33	Espana	2022	Blood	Cebriá-Mendoza et al. 2021[5]
https://www.ncbi.nlm.nih.gov/nucleotide/D90600	D90600	T110	Japan	1999	Plasma	Okamoto et al.1997[10]
https://www.ncbi.nlm.nih.gov/nucleotide/MW467971	MW467971	P9-c1	Espana	2021	Blood	Cebriá-Mendoza et al. 2021[11]
https://www.ncbi.nlm.nih.gov/nucleotide/MZ420571	MZ420571	HPgV_SP20	Espana	2022	Blood	Cebriá-Mendoza et al. 2021[5]
https://www.ncbi.nlm.nih.gov/nucleotide/AF104403	AF104403	European	France	1999	Blood Donor	Charrel et al. 1999[12]
https://www.ncbi.nlm.nih.gov/nucleotide/MZ420572	MZ420572	HPgV_SP29	Espana	2022	Blood	Cebriá-Mendoza et al. 2021[5]
https://www.ncbi.nlm.nih.gov/nucleotide/LT009494	LT009494	56330286	United Kingdom	2016	N.A.	Bonsall et al. 2016[3]
https://www.ncbi.nlm.nih.gov/nucleotide/U45966	U45966	R10291	USA	1996	Plasma	Linnen et al. 1996[6]
https://www.ncbi.nlm.nih.gov/nucleotide/KU685420	KU685420	Cin08	USA	2008	N.A.	Unpublished

REFERENCE

1. Shao L, Shinzawa H, Ishikawa K, Zhang X, Ishibashi M, Misawa H, Yamada N, Togashi H, Takahashi T. Sequence of hepatitis G virus genome isolated from a Japanese patient with non-A-E-hepatitis: amplification and cloning by long reverse transcription-PCR. Biochem Biophys Res Commun. 1996 Nov 21;228(3):785-91. doi: 10.1006/bbrc.1996.1732. PMID: 8941354.

2. Xiang J, Wünschmann S, Schmidt W, Shao J, Stapleton JT. Full-length GB virus C (Hepatitis G virus) RNA transcripts are infectious in primary CD4-positive T cells. J Virol. 2000 Oct;74(19):9125-33. doi: 10.1128/jvi.74.19.9125-9133.2000. PMID: 10982359; PMCID: PMC102111.

3. Bonsall D, Gregory WF, Ip CL, Donfield S, Iles J, Ansari MA, Piazza P, Trebes A, Brown A, Frater J, Pybus OG, Goulder P, Klenerman P, Bowden R, Gomperts ED, Barnes E, Kapoor A, Sharp CP, Simmonds P. Evaluation of Viremia Frequencies of a Novel Human Pegivirus by Using Bioinformatic Screening and PCR. Emerg Infect Dis. 2016 Apr;22(4):671-8. doi: 10.3201/eid2204.151812. PMID: 26982117; PMCID: PMC4806942.

4. George SL, Xiang J, Stapleton JT. Clinical isolates of GB virus type C vary in their ability to persist and replicate in peripheral blood mononuclear cell cultures. Virology. 2003 Nov 25;316(2):191-201. doi: 10.1016/s0042-6822(03)00585-3. PMID: 14644602.

5. Cebriá-Mendoza M, Bracho MA, Arbona C, Larrea L, Díaz W, Sanjuán R, Cuevas JM. Exploring the Diversity of the Human Blood Virome. Viruses. 2021 Nov 21;13(11):2322. doi: 10.3390/v13112322. PMID: 34835128; PMCID: PMC8621239.

6. Linnen J, Wages J Jr, Zhang-Keck ZY, Fry KE, Krawczynski KZ, Alter H, Koonin E, Gallagher M, Alter M, Hadziyannis S,Karayiannis P, Fung K, Nakatsuji Y, Shih JW, Young L, Piatak M Jr, Hoover C, Fernandez J, Chen S, Zou JC, Morris T, Hyams KC, Ismay S, Lifson JD, Hess G, Foung SK, Thomas H, Bradley D, Margolis H, Kim JP. Molecular cloning and disease association of hepatitis G virus: a transfusion-transmissible agent. Science. 1996 Jan 26;271(5248):505-8. doi: 10.1126/science.271.5248.505. PMID: 8560265.

7. Jordier F, Deligny ML, Barré R, Robert C, Galicher V, Uch R, Fournier PE, Raoult D, Biagini P. Human pegivirus isolates characterized by deep sequencing from hepatitis C virus-RNA and human immunodeficiency virus-RNA-positive blood donations, France. J Med Virol. 2019 Jan;91(1):38-44. doi: 10.1002/jmv.25290. Epub 2018 Sep 24. PMID: 30133782.

8. Takahashi K, Hijikata M, Aoyama K, Hoshino H, Hino K, Mishiro S. Characterization of GBV-CHGV viral genome: comparison among different isolates for a ~ 2 kb-sequence that covers entire E1 and most of 5′UTR and E2,International

Hepatology Communications,Volume 6, Issue 5,1997,Pages 253-263,ISSN 0928-4346.doi://doi.org/10.1016/S0928-4346(97)00355-1.

9. Bukh J, Kim JP, Govindarajan S, Apgar CL, Foung SK, Wages J Jr, Yun AJ, Shapiro M, Emerson SU, Purcell RH. Experimental infection of chimpanzees with hepatitis G virus and genetic analysis of the virus. J Infect Dis. 1998 Apr;177(4):855-62. doi: 10.1086/515255. PMID: 9534956.

10. Okamoto H, Nakao H, Inoue T, Fukuda M, Kishimoto J, Iizuka H, Tsuda F, Miyakawa Y, Mayumi M. The entire nucleotide sequences of two GB virus C/hepatitis G virus isolates of distinct genotypes from Japan. J Gen Virol. 1997 Apr;78 (Pt 4):737-45. doi: 10.1099/0022-1317-78-4-737. PMID: 9129645.

11. Cebriá-Mendoza M, Arbona C, Larrea L, Díaz W, Arnau V, Peña C, Bou JV, Sanjuán R, Cuevas JM. Deep viral blood metagenomics reveals extensive anellovirus diversity in healthy humans. Sci Rep. 2021 Mar 25;11(1):6921. doi: 10.1038/s41598-021-86427-4. PMID: 33767340; PMCID: PMC7994813.

12. Charrel RN, Attoui H, De Micco P, de Lamballerie X. The complete coding sequence of a European isolate of GB-C/hepatitis G virus. Biochem Biophys Res Commun. 1999 Feb 16;255(2):432-7. doi: 10.1006/bbrc.1999.0205. PMID: 10049726.
