# Supplementary material for: Pegivirus Detection in Cerebrospinal Fluid from Patients with Central Nervous System Infections of Unknown Etiology in Brazil by Viral Metagenomics
Source: Microorganisms. 2023 Dec 22;12(1):19. doi: 10.3390/microorganisms12010019 (PMC10818654; doi:10.3390/microorganisms12010019)
Supplement: Supplementary file 1 [file microorganisms-12-00019-s001.zip › Supplementary Table S2 Pegivirus Poliprot GenotypeR1.pdf]

Supplementary Table S2. Strain references selected for HPgV genotypes classification

Weblink	NCBI accession number	ID-sequence	HPgV genotype	Country	Year	Sample	Reference
-	OR639931	IAL-425/BRA/SP/2019	1	Brazil	2019	Cerebrospinal Fluid	This study
https://www.ncbi.nlm.nih.gov/nucleotide/D90600	D90600	T110	2	Japan	1999	Plasma	Okamoto et al.1997[1]
https://www.ncbi.nlm.nih.gov/nucleotide/U45966	U45966	R10291	2	USA	1996	Plasma	Linnen et al. 1996[2]
https://www.ncbi.nlm.nih.gov/nucleotide/NC_001710	NC_001710	2700757	2	USA	1996	Plasma	Linnen et al. 1996[2]
https://www.ncbi.nlm.nih.gov/nucleotide/AF309966	AF309966	PEI	2	Germany	2000	Clone	Unpublished
https://www.ncbi.nlm.nih.gov/nucleotide/AF121950	AF121950	Iowan	2	USA	2000	Blood donors	Xiang et al. 2000[3]
https://www.ncbi.nlm.nih.gov/nucleotide/U63715	U63715	GBV-C(EA)	2	East African	1996	Serum	Erker et al. 1996[4]
https://www.ncbi.nlm.nih.gov/nucleotide/AB003291	AB003291	CG12LC	1	Japan	1999	Blood donors	Takahashi et al. 1997[5]
https://www.ncbi.nlm.nih.gov/nucleotide/AY949771	AY949771	D50	1	South Africa	2005	Serum	Muerhoff et al. 2005[6]
https://www.ncbi.nlm.nih.gov/nucleotide/AB013500	AB013500	HGV-GA128	1	Ghana	2006	N.A.	Saito et al. 1999[7]
https://www.ncbi.nlm.nih.gov/nucleotide/AB021287	AB021287	HGV-MY14	4	Myanmar	2000	Serum	Naito et al. 2000[8]
https://www.ncbi.nlm.nih.gov/nucleotide/AB018667	AB018667	HGV-VT48	4	Vietnam	2000	Serum	Naito et al. 2000[8]
https://www.ncbi.nlm.nih.gov/nucleotide/AB003292	AB003292	G05BD	6	Japan	2007	N.A.	Takahashi et al. 1997[5]
https://www.ncbi.nlm.nih.gov/nucleotide/HQ331234	HQ331234	DL185	7	China	2011	Serum	Feng et al. 2011[9]
https://www.ncbi.nlm.nih.gov/nucleotide/HQ331231	HQ331231	KY111	7	China	2011	Serum	Feng et al. 2011[9]
https://www.ncbi.nlm.nih.gov/nucleotide/U94695	U94695	HGVCN	3	China	2003	Serum	Wang et al. 1997[10]
https://www.ncbi.nlm.nih.gov/nucleotide/AF006500	AF006500	HGV-GD	3	China	1997	N.A.	Unpublished
https://www.ncbi.nlm.nih.gov/nucleotide/AB003288	AB003288	BG1HC	3	Japan	1999	Plasma/Serum	Takahashi et al. 1997[5]
https://www.ncbi.nlm.nih.gov/nucleotide/D87711	D87711	K1789	5	Japan	1998	serum	Katayama ET AL. 1998[11]

REFERENCES

1. Okamoto H, Nakao H, Inoue T, Fukuda M, Kishimoto J, Iizuka H, Tsuda F, Miyakawa Y, Mayumi M. The entire nucleotide sequences of two GB virus C/hepatitis G virus isolates of distinct genotypes from Japan. J Gen Virol. 1997 Apr;78 (Pt 4):737-45. doi: 10.1099/0022-1317-78-4-737. PMID: 9129645.

2. Linnen J, Wages J Jr, Zhang-Keck ZY, Fry KE, Krawczynski KZ, Alter H, Koonin E, Gallagher M, Alter M, Hadziyannis S, Karayiannis P, Fung K, Nakatsuji Y, Shih JW, Young L, Piatak M Jr, Hoover C, Fernandez J, Chen S, Zou JC, Morris T, Hyams KC, Ismay S, Lifson JD, Hess G, Foung SK, Thomas H, Bradley D, Margolis H, Kim JP. Molecular cloning and disease association of hepatitis G virus: a transfusion-transmissible agent. Science. 1996 Jan 26;271(5248):505-8. doi: 10.1126/science.271.5248.505. PMID: 8560265.

3. Xiang J, Wünschmann S, Schmidt W, Shao J, Stapleton JT. Full-length GB virus C (Hepatitis G virus) RNA transcripts are infectious in primary CD4-positive T cells. J Virol. 2000 Oct;74(19):9125-33. doi: 10.1128/jvi.74.19.9125-9133.2000. PMID: 10982359; PMCID: PMC102111.

4. Erker JC, Simons JN, Muerhoff AS, Leary TP, Chalmers ML, Desai SM, Mushahwar IK. Molecular cloning and characterization of a GB virus C isolate from a patient with non-A-E hepatitis. J Gen Virol. 1996 Nov;77 (Pt 11):2713-20. doi: 10.1099/0022-1317-77-11-2713. PMID: 8922464.

5. Takahashi K, Hijikata M, Aoyama K, Hoshino H, Hino K, Mishiro S. Characterization of GBV-CHGV viral genome: comparison among different isolates for a ~ 2 kb-sequence that covers entire E1 and most of 5′UTR and E2,International Hepatology Communications,Volume 6, Issue 5,1997,Pages 253-263,ISSN 0928-4346.doi://doi.org/10.1016/S0928-4346(97)00355-1.

6. Muerhoff AS, Leary TP, Sathar MA, Dawson GJ, Desai SM. African origin of GB virus C determined by phylogenetic analysis of a complete genotype 5 genome from South Africa. J Gen Virol. 2005 Jun;86(Pt 6):1729-1735. doi: 10.1099/vir.0.80854-0. PMID: 15914851.

7. Saito T, Ishikawa K, Osei-Kwasi M, Kaneko T, Brandful J, Nuvor V, Aidoo S, Ampofo W, Apeagyei F, Ansah JE, Adu-Sarkodie Y, Nkrumah FK, Abe K ,Prevalence of hepatitis G virus and characterization of viral genome.Hepatology Research,Volume 13, Issue 3,1999,Pages 221-231,ISSN 1386-6346,https://doi.org/10.1016/S1386-6346(98)00095-3.

8. Naito H, Hayashi S, Abe K. The entire nucleotide sequence of two hepatitis G virus isolates belonging to a novel genotype: isolation in Myanmar and Vietnam. J Gen Virol. 2000 Jan;81(Pt 1):189-94. doi: 10.1099/0022-1317-81-1-189. PMID: 10640557.

9. Feng Y, Zhao W, Feng Y, Dai J, Li Z, Zhang X, Liu L, Bai J, Zhang H, Lu L, Xia X. A novel genotype of GB virus C: its identification and predominance among injecting drug users in Yunnan, China. PLoS One. 2011;6(10):e21151. doi: 10.1371/journal.pone.0021151. Epub 2011 Oct 6. PMID: 21998624; PMCID: PMC3188531.

10. Wang HL, Jin DY. Prevalence and genotype of hepatitis G virus in Chinese professional blood donors and hepatitis patients. J Infect Dis. 1997 May;175(5):1229-33. doi: 10.1086/593676. PMID: 9129092.

11. Katayama K, Kageyama T, Fukushi S, Hoshino FB, Kurihara C, Ishiyama N, Okamura H, Oya A. Full-length GBV-C/HGV

genomes from nine Japanese isolates: characterization by comparative analyses. Arch Virol. 1998;143(6):1063-75. doi: 10.1007/s007050050356. PMID: 9687865.
